# Complete Endotracheal Tube Obstruction Due to Highly Viscous Secretions Immediately After Tracheal Intubation: A Case Report

**DOI:** 10.7759/cureus.78463

**Published:** 2025-02-03

**Authors:** Takashi Saga, Koji Sato, Yukitoshi Niiyama

**Affiliations:** 1 Department of Anesthesiology and Intensive Care Medicine, Akita University Graduate School of Medicine, Akita, JPN

**Keywords:** advanced age, bronchoscopy, capnographic waveform, endotracheal tube obstruction, highly viscous secretions

## Abstract

Obstruction of the endotracheal tube (ETT) from secretions can cause severe respiratory distress and potentially life-threatening complications; therefore, prompt decisions and management are needed. Cases of ETT obstruction have been observed in patients who have been ventilated for extended periods but are rare in patients immediately after endotracheal intubation. Herein, we present a rare case of ETT obstruction after endotracheal intubation. A 90-year-old male was scheduled for open reduction and internal fixation (ORIF) under general anesthesia for a femoral fracture. The patient had a history of hypertension, chronic atrial fibrillation, chronic heart failure, and esophageal cancer. After the induction of anesthesia, viscous secretions were observed in the oral cavity when the larynx was exposed. Secretions were removed using Magill forceps and aspiration. An ETT was placed using a videolaryngoscope, and mechanical ventilation was initiated. Twelve minutes after intubation, although the airway pressure alarm did not sound, the capnographic waveforms disappeared; therefore, manual ventilation with 100% oxygen was initiated. However, ventilation was not possible. Suspecting obstruction of the ETT by secretions, tracheal suction was attempted; however, the suction catheter could not be inserted. The patient developed bradycardia and hypotension. Chest compressions were initiated, and the ETT was removed. Ventilation was possible after intubation using a new ETT, and the circulatory dynamics stabilized. The previous ETT was examined and found completely obstructed by highly viscous secretions. The patient was able to undergo the ORIF upon stabilization. The procedure was performed without complications or postoperative neurological deficits. Several potential causes of the ETT obstruction immediately after intubation were considered: upward migration of the bronchial secretions, progressive occlusion by secretions adhered to the ETT lumen, and a combination of these patterns. Capnographic waveform analysis played a critical role in identifying the obstruction, with key findings including the disappearance of the waveform. In cases where heavy secretions are anticipated, preparing a bronchoscope during anesthesia induction and careful monitoring with capnography are essential for the early detection and management of ETT obstruction.

## Introduction

Obstruction of the endotracheal tube (ETT) due to secretions can cause severe respiratory distress and potentially life-threatening complications; therefore, prompt decision-making and management are needed. Rapid differential diagnosis from other conditions that may cause ventilatory insufficiency, such as dislodgement or displacement of the ETT, obstruction due to biting, pneumothorax, chest wall rigidity, and equipment-related malfunctions, is particularly important [1,2]. Changes in the capnographic waveform can be useful in identifying obstructions.

Cases wherein the lumen of the ETT has become obstructed by secretions have been reported in patients with long-term intubation [3]; however, reports of complete occlusion of the ETT within a short period following tracheal intubation are rare. Herein, we present the case of a patient with complete ETT obstruction caused by highly viscous secretions immediately after endotracheal intubation.

This case was previously presented as a poster presentation at the 11th Annual Satellite (Hokkaido-Tohoku) meeting of the Japanese Society of Anesthesiologists (September 2021, WEB).

## Case presentation

Clinical background

A 90-year-old male patient (height, 140 cm; weight, 43 kg) diagnosed with a femoral subtrochanteric fracture was scheduled for open reduction and internal fixation (ORIF). Past medical history included hypertension, chronic atrial fibrillation, chronic heart failure, and esophageal cancer. Other history included smoking until the age of 72. The patient had previously undergone radical surgery for esophageal cancer under general anesthesia. The patient exhibited dementia and was unable to communicate.

**Figure 1:**
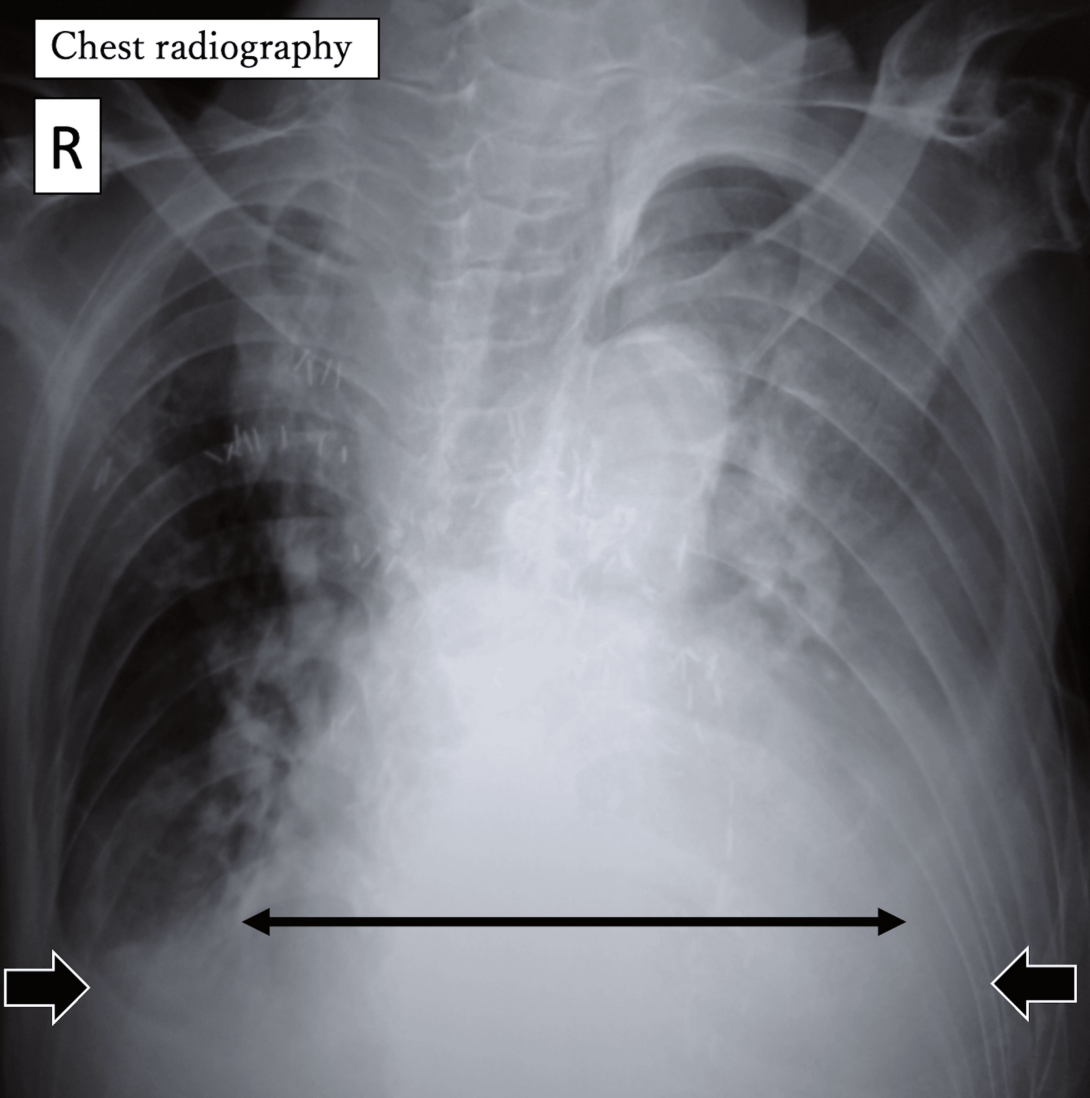
Chest radiography Preoperative chest radiography revealed marked cardiomegaly (↔︎), with a cardiothoracic ratio (CTR) of 70%, and bilateral pleural effusions (➡︎). R, right

At admission, blood tests revealed anemia with a hemoglobin level of 9.6 g/dL, malnutrition with an albumin level of 2.4 g/dL, and mild dehydration with a blood urea nitrogen (BUN) level of 27.0 mg/dL and creatinine level of 0.72 mg/dL (Table 1). Arterial blood gas analysis (room air) showed hypoxia and hypercapnia (pH 7.430, PaO_2_ 64.1 mmHg, PaCO_2_ 55.6 mmHg, and HCO_3_^-^ 36.3 mmol/L) (Table 2), suggesting the potential presence of chronic obstructive pulmonary disease. Chest radiography revealed marked cardiomegaly, with a cardiothoracic ratio (CTR) of 70%, and bilateral pleural effusions (Figure 1). Preoperative electrocardiography revealed atrial fibrillation with a heart rate of 80 beats per minute (bpm) and no significant ST changes. As the patient was unable to communicate, respiratory function tests were not performed.

**Table 1:** Preoperative laboratory data Reference ranges are shown in parentheses. AST, aspartate aminotransferase; ALT, alanine aminotransferase

Laboratory Data	Value
White blood cell count (3.3-8.6 × 10^9^/L)	9.9
Hemoglobin (13.7-16.8 g/dL)	9.6
Platelet count (15.8-34.8 × 10^3^/L)	12.6
Albumin (4.1-5.1 g/dL)	2.4
Blood urea nitrogen (8-20 mg/dL)	27.0
Creatinine (0.65-1.07 mg/dL)	0.72
Total bilirubin (0.4-1.5 mg/dL)	0.8
AST (13-30 U/L)	29
ALT (10-42 U/L)	25
Sodium (138-145 mmol/L)	144
Potassium (3.6-4.8 mmol/L)	3.7
Chloride (101-108 mmol/L)	100

**Table 2:** Preoperative arterial blood gas analysis (room air) *The age-adjusted normal value for PaO₂ is calculated using the formula "102 - age/3," which yields 72 mmHg for this patient. Reference ranges are shown in parentheses. PaO_2_, partial pressure of oxygen; PaCO_2_, partial pressure of carbon dioxide; HCO_3_^-^, bicarbonate

Arterial Blood Gas Analysis (Room Air)	Value
pH (7.35-7.45)	7.43
PaO_2_ (80-100 mmHg)*	64.1
PaCO_2_ (35-45 mmHg)	55.6
HCO_3_^-^ (20.0-26.0 mmol/L)	36.3
Base excess (-3.0-3.0 mmol/L)	10.8
Lactate (4.5-18.0 mg/dL)	8.0

Operation and anesthesia

ORIF was planned (seven days post femur fracture). Upon the patient's arrival at the operating room, electrocardiography, pulse oximetry, and noninvasive blood pressure monitoring were conducted. Prior to inducing anesthesia, an arterial pressure line was inserted in the left radial artery. Baseline parameters were as follows: heart rate, 80 bpm; blood pressure, 90/52 mmHg; and SpO_2_, 99% (room air).

General anesthesia was administered combined with femoral and lateral femoral cutaneous nerve blocks. After preoxygenation, anesthesia was induced with remifentanil at 0.15 mcg/kg/minute and propofol 20 mg. Rocuronium 40 mg was administered for muscle relaxation. Mask ventilation was administered without difficulty, and the larynx was exposed using a videolaryngoscope. Inspection of the oral cavity revealed a large amount of highly viscous secretions. We attempted suctioning using a 14 French suction catheter; however, the high viscosity of the secretions made suctioning difficult. Therefore, the visible secretions were removed using Magill forceps. Thereafter, an ETT (internal diameter, 8.0 mm) was inserted into the trachea and secured 20 cm from the right corner of the mouth. The ventilator was set to volume control mode with a tidal volume of 320 mL, a respiratory rate of 12 breaths per minute, a positive end-expiratory pressure (PEEP) of 5 cmH_2_O, and an inspiratory-to-expiratory (I:E) ratio of 1:2, resulting in a peak airway pressure of 20 cmH_2_O. The peak airway pressure alarm was set at 40 cmH_2_O. The capnographic waveform indicated an obstructive pattern. Secretions noted within the ETT were suctioned using a suction catheter, and the lumen of the ETT was confirmed to be patent. Anesthesia was maintained using air, oxygen, sevoflurane, and remifentanil.

**Figure 2:**
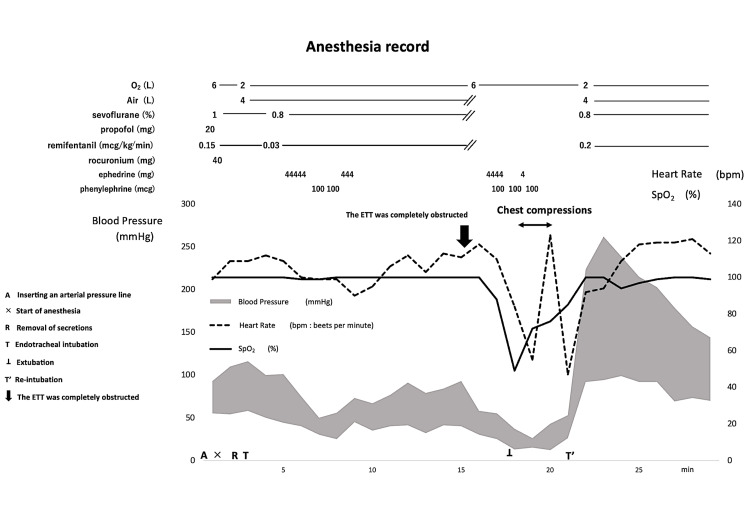
Anesthesia documentation from the initiation of anesthesia until reintubation An arterial line was inserted (A) prior to initiating general anesthesia (×). Oral secretions were removed using Magill forceps (R), and the ETT was placed via tracheal intubation (T). Twelve minutes after intubation, the capnographic waveforms disappeared; the ETT was obstructed (➡︎). Bradycardia developed, and chest compressions were performed for approximately three minutes (↔︎). The ETT was removed (⊥), followed by mask ventilation, and reintubation was performed (T’). Subsequently, the vital signs significantly improved. ETT, endotracheal tube

Following intubation, the patient developed hypotension, which was managed with vasopressor administration of phenylephrine or ephedrine. Twelve minutes after intubation, although the airway pressure alarm did not sound, the capnographic waveform disappeared. Manual ventilation with 100% oxygen was attempted, but ventilation was impossible. The bag compliance was low, and the capnographic waveform still was not observed. The integrity of the anesthesia was confirmed to ensure that the inability to ventilate was not due to the disconnection or kinking of the anesthesia circuit. Considering that the ETT may have been obstructed by secretions, suctioning was administered; however, the suction catheter could not pass through the ETT. The SpO_2_ dropped to 49%. We immediately removed the ETT, and mask ventilation was initiated. Bradycardia and hypotension gradually developed (55 bpm, 26/15 mmHg), and chest compressions were initiated. Figure 2 shows the course of events during anesthesia.

**Figure 3:**
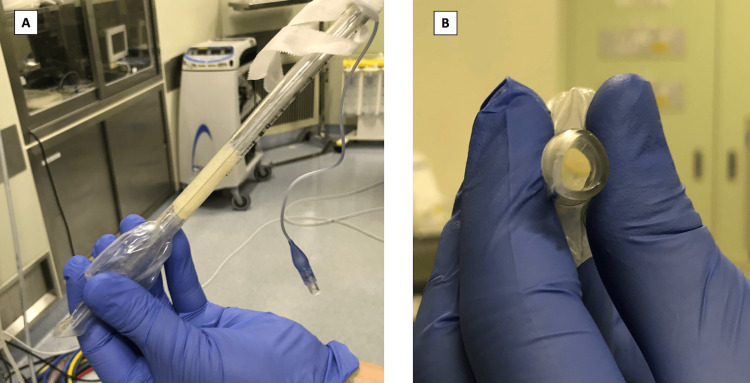
The extubated endotracheal tube (A) ETT viewed from the side; (B) ETT viewed from the tip. The lumen of the ETT was completely obstructed by highly viscous secretions. ETT, endotracheal tube

The examination of the ETT revealed highly viscous secretions adhering to the interior of the lumen, which was completely obstructed (Figure 3). After inserting a new ETT, ventilation was possible, the capnographic waveform normalized, and circulation stabilized. Secretions within the trachea and bronchus were suctioned using a bronchoscope, and no visible secretions remained. Fifteen minutes after reintubation, the arterial blood gas analysis (with FiO_2_ of 0.45) revealed a pH of 7.436, PaCO_2_ 54.5 mmHg, PaO_2_ 91.0 mmHg, and HCO_3_⁻ 26.0 mmol/L. Subsequently, the respiratory and hemodynamic parameters remained stable, allowing surgery to proceed. The duration of the anesthesia was 167 minutes, and the duration of the surgery was 50 minutes. At the end of the anesthesia, the patient exhibited adequate spontaneous breathing and was responsive. Therefore, the patient was extubated and transferred to the intensive care unit. No significant respiratory deterioration or neurological deficits were observed postoperatively.

## Discussion

Cases of complete ETT obstruction after prolonged tracheal intubation have been previously reported; however, ETT obstruction soon after intubation, as in the present case, is rare [4].

Human bronchial mucus is produced by the bronchial and tracheal glands as well as by the goblet cells located in the airway epithelium. The present case involved a patient with a history of chronic heart failure and advanced age; both diagnoses can lead to increased secretions [5]. Additionally, dehydration can contribute to thickness and viscosity caused by the drying of secretions and increase the risk of ETT obstruction. Typically, bronchial secretions are transported proximally by ciliary motility and expiration and are subsequently managed by swallowing or expelled by coughing. Factors such as smoking, lung disease, obesity, advanced age, surgery, and anesthesia can adversely affect these mechanisms [6]. In the present case, advanced age may have contributed to the decreased preoperative secretory clearance capacity. In addition, the patient had a history of radical esophageal cancer surgery. This may have resulted in inadequate clearance of secretions due to poor swallowing function and accumulation of secretions in the oral cavity, leading to asymptomatic aspiration [7]. Moreover, the seven-day interval from the femur injury to surgery may have contributed to the deterioration of the patient's overall condition. Furthermore, manifestations from chronic heart failure may have exacerbated sputum production and impaired the patient's ability to clear respiratory secretions.

**Figure 4:**
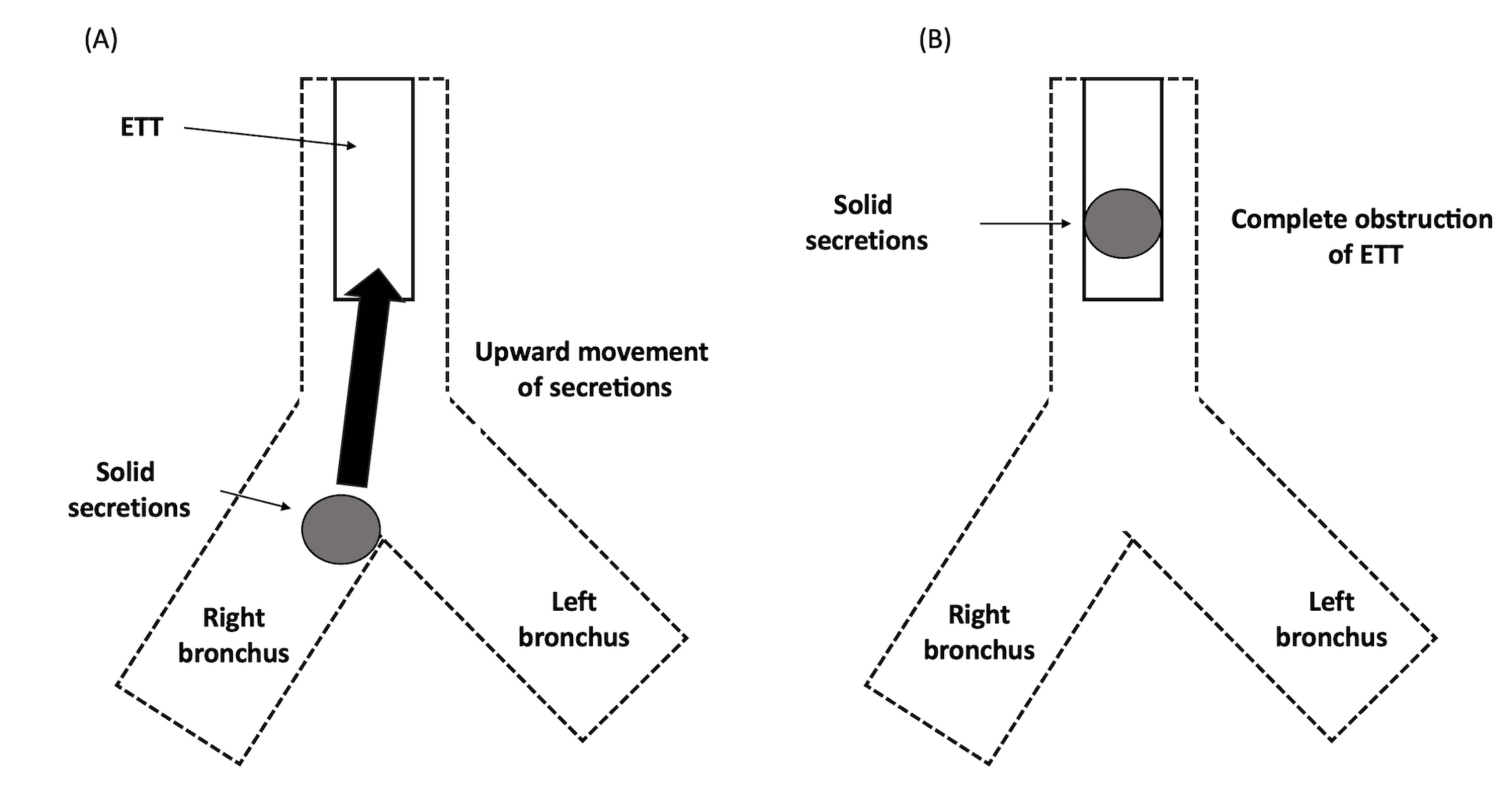
Pattern 1 of endotracheal tube obstruction The dotted lines represent the trachea and bronchus, the box represents the ETT, and the circle represents solid secretions. (A) The lumen of the ETT was patent after the initial intubation, and highly viscous secretions were present in the bronchus. (B) Under certain circumstances, such as in the present case, the secretions can migrate upward into the ETT and cause obstruction. Image created by the authors. ETT, endotracheal tube

We considered three patterns as potential causes of the complete obstruction of the ETT in our patient. Pattern 1 involves highly viscous secretions from the bronchus entering and obstructing the ETT. Figure 4A shows the presence of highly viscous secretions within the bronchi. These secretions may have obstructed the ETT. The upward migration of mucus plugs in the lower airways can cause an ETT obstruction during surgery (Figure 4B) [8]. This phenomenon is more likely to occur during anesthesia induction and is characterized by abrupt movements, apnea, or a sudden cough reflex. In addition, this may occur after changes in patient positioning and often presents with sudden signs of airway obstruction. In the present case, no apparent cough reflex was observed prior to the occurrence of obstruction. However, this phenomenon may have occurred owing to suctioning after tracheal intubation.

**Figure 5:**
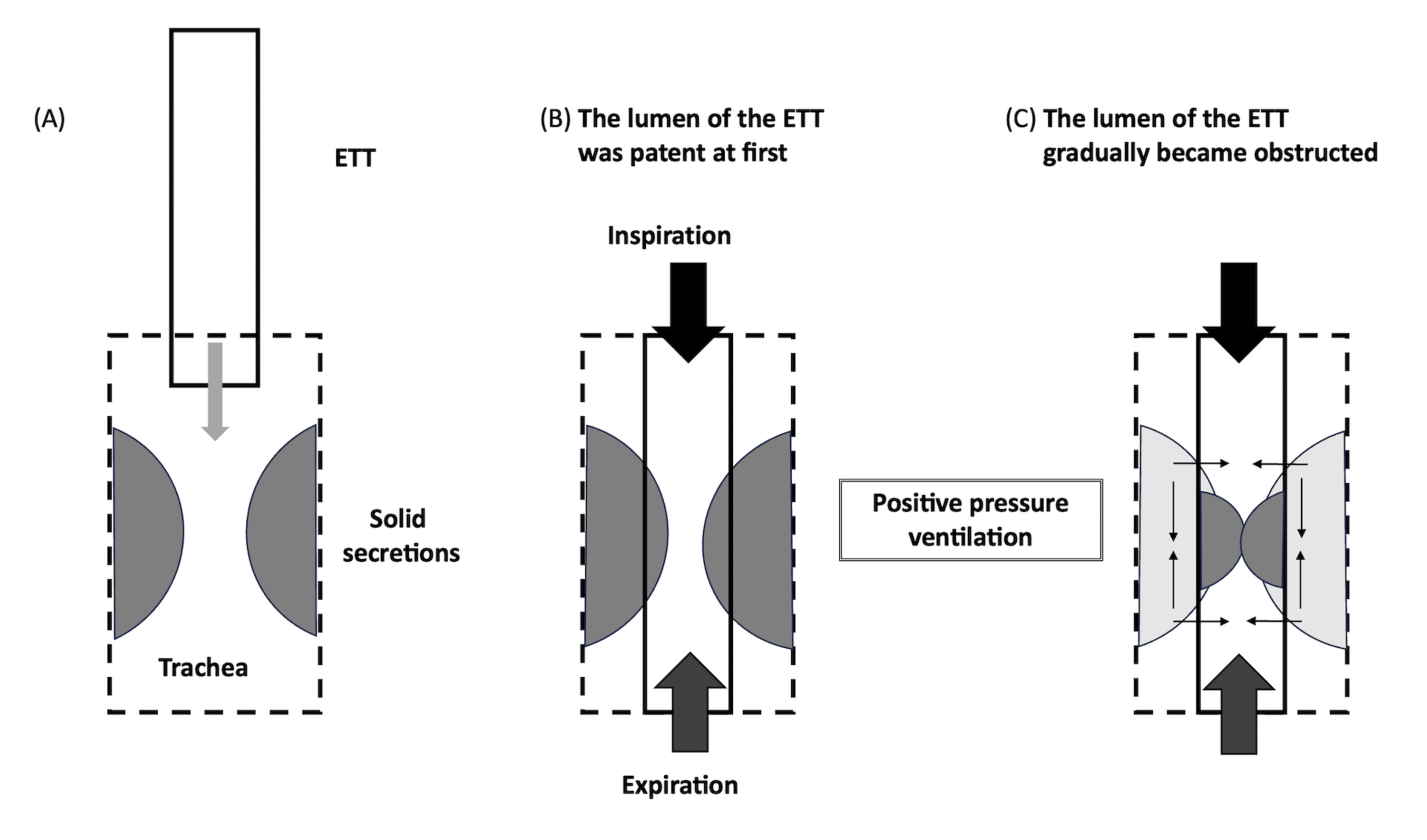
Pattern 2 of endotracheal tube obstruction The dotted line represents the trachea, the box represents the ETT, and the two semicircles represent secretions. (A) Highly viscous secretions that may have adhered to the tracheal wall may have been scraped off by the ETT during intubation. (B) The lumen of the first ETT was initially patent. (C) The lumen of the ETT gradually became obstructed by positive pressure ventilation. Image created by the authors. ETT, endotracheal tube

In pattern 2, secretions adhering to the lumen of the ETT can increase over time and occlude the ETT [9]. Secretions within the ETT may originate from the tracheal secretions that have entered the ETT or that have been scraped from the tracheal wall by the ETT during intubation (Figure 5A). In the present patient, the viscous secretions may have been scraped from the tracheal wall and released into the ETT during intubation. Endotracheal suctioning was possible immediately after intubation; therefore, the lumen of the ETT was likely partially patent (Figure 5B). The mechanism of the subsequent positive pressure ventilation may have "compressed/compacted" the secretions and eventually led to complete occlusion (Figure 5C). The third pattern is a combination of patterns 1 and 2. The viscous secretions present on the tracheal wall that may have been scraped off peripherally by the ETT during intubation may have migrated upwards, causing ETT obstruction. However, in the present case, we were unable to determine the specific pattern and definitively diagnose the underlying mechanism.

If mechanical ventilation becomes difficult, manual ventilation with 100% oxygen must be initiated immediately. Auscultation of breath sounds, confirmation of airway pressure, and observation of the capnographic waveform should be promptly performed to determine the underlying cause(s). If the tracheal tube is completely occluded, the capnographic waveform disappears [10,11]. In the present case, the disappearance of the capnographic waveform played an important role in identifying the cause of the airway complications.

In mechanically ventilated patients, airway obstruction should be suspected when abnormally high peak inspiratory pressures, reduced lung compliance, increased resistance to expansion, and significant discrepancies between measured inspiratory and expiratory tidal volumes are observed [12]. Furthermore, if circulatory failure develops owing to ETT obstruction, extubation followed by reintubation should be performed [12]. In the present case, the airway pressure alarm did not sound. However, the disappearance of the capnographic waveforms, inability to pass a suction catheter, presence of copious secretions before intubation, and absence of other identifiable causes of ventilatory failure led to the consideration of ETT obstruction. When the present patient exhibited circulatory failure, we removed the ETT and intubated using a new ETT. This response is considered an appropriate course of action.

In intubated patients, ETT obstruction can be identified by inserting an appropriately sized suction catheter to assess the patency of the ETT [12]. However, some reports have indicated that endotracheal suctioning alone may not completely remove secretions in the ETT [4]. Preventing complete obstruction by suctioning alone is difficult and may not always be possible. Furthermore, some reports have suggested that complete airway obstruction cannot be entirely ruled out even if a suction catheter passes through an ETT [13]. Thus, excluding the possibility of airway obstruction by relying solely on the passage of a suction catheter is not adequate. Although the lumen of the ETT was likely partially patent immediately after intubation in the present case, the ETT was completely obstructed by secretions within 12 minutes.

Airway obstruction should be considered when large amounts of highly viscous secretions are observed in the oral cavity. Regretfully, upon intubation, we did not conduct bronchoscopy for immediate observation of the trachea. In patients with large amounts of secretions preoperatively, observation of the trachea using a bronchoscope immediately after endotracheal intubation may identify or predict a risk of ETT obstruction.

In the present case, hypoxemia, bradycardia, and hypotension were observed; however, cardiac arrest did not occur. Therefore, we opted to proceed with the ORIF. However, in hindsight, we acknowledge that awakening the patient temporarily from the anesthesia and confirming the absence of neurological deficits to determine stability for surgery may have been the preferred course of action.

## Conclusions

We encountered a case of complete ETT obstruction caused by highly viscous secretions immediately after intubation. If large amounts of highly viscous secretions are anticipated, as in the present case, the potential for ETT obstruction should be considered. In such situations, close monitoring of the capnographic waveform and preemptive bronchoscopic preparation are essential for the rapid detection and management of ETT obstruction.
